# ENDOCRINOLOGY IN THE TIME OF COVID-19: Management of diabetes insipidus and hyponatraemiaThis manuscript is part of a commissioned series of urgent clinical guidance documents on the management of endocrine conditions in the time of COVID-19. This clinical guidance document underwent expedited open peer review by Joe Verbalis (Georgetown University, USA), Jens Otto Jørgensen (Aarhus University. Denmark), Stefan Bilz (Cantonal Hospital St.Gallen, Switzerland) and Georg Lindner (Inselspital, Switzerland)

**DOI:** 10.1530/EJE-20-0338

**Published:** 2020-07-01

**Authors:** Mirjam Christ-Crain, Ewout J Hoorn, Mark Sherlock, Chris J Thompson, John A H Wass

**Affiliations:** 1 Division of Endocrinology, Diabetes and Metabolism, Department of Internal Medicine and Department of Clinical Research, University Hospital Basel, University of Basel, Basel, Switzerland; 2 Division of Nephrology and Transplantation, Department of Internal Medicine, Erasmus MC, University Medical Center Rotterdam, Rotterdam, The Netherlands; 3 Academic Department of Endocrinology, Beaumont Hospital/RCSI Medical School, Dublin, Ireland; 4 Department of Endocrinology, Oxford Centre for Diabetes, Endocrinology and Metabolism, Churchill Hospital, Oxford, UK

## Abstract

COVID-19 has changed the nature of medical consultations, emphasizing virtual patient counseling, with relevance for patients with diabetes insipidus (DI) or hyponatraemia. The main complication of desmopressin treatment in DI is dilutional hyponatraemia. Since plasma sodium monitoring is not always possible in times of COVID-19, we recommend to delay the desmopressin dose once a week until aquaresis occurs allowing excess retained water to be excreted. Patients should measure their body weight daily. Patients with DI admitted to the hospital with COVID-19 have a high risk for mortality due to volume depletion. Specialists must supervise fluid replacement and dosing of desmopressin. Patients after pituitary surgery should drink to thirst and measure their body weight daily to early recognize the development of the postoperative syndrome of inappropriate antidiuresis (SIAD). They should know hyponatraemia symptoms. The prevalence of hyponatraemia in patients with pneumonia due to COVID-19 is not yet known, but seems to be low. In contrast, hypernatraemia may develop in COVID-19 patients in ICU, from different multifactorial reasons, for example, due to insensible water losses from pyrexia, increased respiration rate and use of diuretics. Hypernatraemic dehydration may contribute to the high risk of acute kidney injury in COVID-19. IV fluid replacement should be administered with caution in severe cases of COVID-19 because of the risk of pulmonary oedema.

## Introduction

Patients with pre-existing endocrine conditions may be vulnerable to perturbations in plasma sodium in more severe cases of COVID-19. None of the published reports so far have reported a higher prevalence of dysnatraemia in COVID-19 (1, 2, 3), even in elderly cohorts (4). However, patients with central diabetes insipidus (DI) or pre-existing hyponatraemia may be at risk of more severe, life-threatening dysnatraemia.

This article is intended to advise how endocrinologists can still optimally care for ambulatory patients with central DI and hyponatraemia, where regular physical consultations and biochemical assessments are not possible. Furthermore, we provide guidance on management of these patients when they are admitted to the hospital with severe COVID-19.

## Diabetes insipidus

DI is a rare disease (5), characterized by hypotonic polyuria and polydipsia (6). The differential diagnosis of DI involves the distinction between primary forms (central DI) or nephrogenic DI, and secondary forms, where polyuria results from primary polydipsia (7). Treatment of DI consists of fluid administration in case of dehydration, and in cases of central DI, desmopressin as hormone replacement for the absent vasopressin (8).

### How to manage patients with an established diagnosis of diabetes insipidus in the time of COVID-19

#### Management of patients with central DI in the outpatient setting

- In the majority of cases with central DI, osmoregulated thirst is intact, and oral fluid intake accurately compensates for urinary and insensible water losses. Even prior to treatment with desmopressin, patients are therefore typically eunatraemic. To reduce the symptoms of polyuria and polydipsia, desmopressin is given orally or intranasally; the major complication of desmopressin therapy is hyponatraemia. A retrospective review has shown that 27% of central DI patients show mild hyponatraemia (131–134 mmol/L) on routine electrolyte testing and 15% develop more severe hyponatraemia (≤130 mmol/L), over long-term follow-up (9). Hyponatraemia develops when the antidiuretic effects of continuous desmopressin therapy prevent free water excretion, even with normal fluid intakes. This can be prevented by delaying doses of desmopressin to allow regular aquaresis, but regular electrolyte checks are recommended during initiation of therapy. Annual electrolyte checking is recommended for long-term follow-up, though more frequent monitoring is needed where hyponatraemia episodes are more frequent.
- The COVID-19 pandemic has limited the accessibility of blood testing, and the **priority of routine treatment of central DI should be to avoid hyponatraemia. This emphasises the importance of delaying desmopressin doses,** to allow regular periods of free water clearance, so that excess water intake does not lead to dilutional hyponatraemia. This can be achieved by recommending that the patient delays a dose of desmopressin once or twice per week, until an aquaresis occurs. Some patients regularly delay each dose until they begin to feel polyuric, as they are aware that they otherwise feel bloated by fluid retention. Alternatively, especially in patients known to experience recurrent hyponatraemia, one dose each week can be entirely omitted, though significant polyuria and social disruption may occur.

#### Acute infection with suspected or confirmed COVID-19

- Although patients with central DI should be no more vulnerable to COVID-19 than the rest of the population, **those DI patients who develop respiratory complications of COVID-19 are at significantly increased risk of dysnatraemia**.
- Epidemiological data show that hypernatraemia is rare in ambulatory patients with DI; in contrast, the rate of hypernatraemia during hospital admission is significant (9). The aetiology of this hypernatraemia is multifactorial; if cognition is attenuated by critical illness, fluid intake may be reduced, and if the patient is vomiting, oral desmopressin intake may be difficult. Most hospital studies report increased mortality in intensive care units associated with hypernatraemia (10), and it is a poor prognostic sign in patients who develop DI following head injury (11). In addition, data from a nationwide Swiss cohort study showed an increased mortality rate in complex hypopituitary patients with central DI admitted to hospital, compared with hypopituitary patients without DI, consistent with vulnerability of DI patients to develop hypernatraemic dehydration in the context of severe illness (Kutz *et al*., unpublished data). The propensity to develop hypernatraemia during hospital admission is particularly marked in patients with adipsic DI (9), and this subgroup of DI patients have been documented to develop severe hypernatraemia (12), which may be complicated by thrombotic episodes (13). Patients with **adipsic DI** may also have hypothalamic obesity, and there is accumulating evidence that obesity *per se* may be an important risk factor for poor outcome in patients with Covid-19. Thus, these patients **may be amongst the most vulnerable and should have a very close follow-up in case of any superimposed illness.**
- In addition, since patients with nephrogenic DI do not respond to desmopressin, they are particularly prone to severe hypernatraemia with inadequate fluid replacement.
- In 2018, the Society for Endocrinology (SfE) published guidelines on in-hospital management of central DI (14). This report was prompted by a study of patients hospitalized with central DI, which showed that desmopressin treatment had been missed or delayed in 88% of admissions, and that 35% of patients consequently developed dysnatraemia (15). This was attributed to a lack of understanding of the critical nature of desmopressin amongst clinical staff (16). In this context it is very important that patients themselves are empowered with a knowledge of their condition and the essential difference between DI and diabetes mellitus which has caused confusion with serious consequences at the time of admission. The results of these guidelines have generated a sensible basis for management of DI when patients are admitted to hospital.
- The key practice points in the SfE in 2018 guidelines are particularly valid for DI patients with COVID-19: **most importantly, all patients with central DI admitted to the hospital with COVID-19 should be managed in consultation with endocrinology advice. In addition, a careful prescribing alert system for all patients treated with desmopressin is recommended, to reduce prescribing errors, and to ensure that essential desmopressin therapy is maintained.**
- In patients with mild COVID-19 cold symptoms, who are alert and able to drink, it may be advisable to prescribe oral rather than nasal desmopressin due to the limited absorption from congested nasal passages. As COVID-19 is characterised by persistent fever and tachypnoea, insensible water losses are likely to be substantially increased; ordinarily, osmotically stimulated drinking should generate fluid intake sufficient to make up for insensible losses, but if cognitive function is impaired by fever, hypoxia or sepsis, i.v. fluids may be required.
- **In patients with severe COVID illness, desmopressin should be given parenterally,** usually with a starting dose of 0.5 µg.
  The i.v. route is generally preferred because it obviates concerns about absorption and has the same total duration of action as the other parenteral routes. Prompt reduction in urine output should occur, and the antidiuretic effect generally lasts for 6–12 h. Urine osmolality and urine volume should be monitored to ascertain whether the dose was effective, and the plasma sodium measured at frequent intervals (every 2–4 h) to ensure improvement of hypernatraemia. Since arterial blood gas point of care testing for sodium may be the main laboratory estimation available in some hospitals (particularly ‘field hospitals’), sodium measurement can be done using this method instead of sending it to the laboratory.
- Initial signals show that **hypernatraemia in COVID-19 patients (without central DI) in intensive care units may be common;** excessive insensible water losses from the constant pyrexia and increased respiration rate may play a major aetiological role in this as well as the need in some patients for significant diuretic use or conservative fluid regimens in order to aid oxygenation. **Hypernatraemic dehydration may therefore rapidly develop in patients with DI,** in particular, those who need diuretic therapy or in whom desmopressin is withheld or delayed. If diuresis is necessary, the optimum management of these patients should be a collaboration between intensive care and endocrinology.
- **In patients with hypovolaemic shock due to COVID-19 restoration of blood volume with i.v. 0.9% sodium chloride is preferable, even if there is hypernatraemia. In the absence of hypovolaemic shock, patients with DI and severe dehydration should be treated with hypotonic fluids**, either enterally (using water or milk) or, if necessary, intravenously (using 5% dextrose in water). Hypotonic fluids should be administered as an intravenous infusion, with the rate adjusted to exceed the hourly urine output and reverse the calculated total body water deficit. The usual aim is to provide just enough water to safely normalize serum sodium at a rate of <0.5 mmol/L per h (<10–12 mmol/L per day) (17). However, the issue is complicated in COVID-19 by other priorities of management in severe disease. Advanced cardiorespiratory complications of COVID-19 are characterized mainly by acute respiratory distress syndrome (ARDS), with significant pulmonary oedema, and sometimes by acute kidney injury (18). Patients who are seriously ill need diuretic therapy to support the lungs or renal replacement therapy if there is concurrent acute kidney injury with reduced urine output. **Intensive care colleagues may be reluctant to reverse hypernatraemia with i.v. hypotonic fluids, because of the risk of pulmonary oedema**. Of note, however, hypotonic fluids are less likely to cause extracellular volume expansion since two-thirds of the administered fluid is distributed intracellularly.
- In contrast to other critically ill patients, so far, hypernatraemia has not been highlighted as a risk factor for severe mortality in COVID-19 (3), possibly due to the only short time period of observation. However, **it may be that endocrinologists have to accept mild hypernatraemia (<155 mmol/L) as the price of preventing pulmonary oedema**. In patients with central DI we will have a major role in ensuring that severe hypernatraemia, which compromises recovery, does not occur, and multidisciplinary input is needed to discuss the individual merits and contingencies of treatment in DI patients with COVID-19.
- It is important to stress that, as hypernatraemic dehydration is associated with a hypercoaguable state, the risk of venous thrombosis, and pulmonary embolism, is substantial, particularly in an immobile patient (19). We therefore **recommend the routine prescription of prophylactic s.c. low-molecular-weight heparin** during episodes of hypernatraemic dehydration, until eunatraemia is restored. This is particularly important since pulmonary embolism is emerging as one of the factors associated with mortality in COVID-19 patients. Low-molecular-weight heparin is also recommended in patients with hypercoaguable states in COVID-19 (20).
- As the majority of patients with postoperative or post-traumatic central DI also have ACTH deficiency, concomitant stress dose corticosteroids are essential during COVID-19 infection.

## Hyponatraemia

Hyponatraemia (plasma sodium <135 mmol/L) is the most common electrolyte disorder in clinical practice (21). It is divided into euvolaemic, hypovoalemic, and hypervolaemic hyponatraemia, each of which is treated differently (22, 23). The syndrome of inappropriate antidiuresis (SIAD) is the commonest cause of hyponatraemia (21, 24).

### How to manage patients with hyponatraemia in the time of COVID-19

#### Management of patients with hyponatraemia in the routine endocrine practice

- SIAD is seen by endocrinologists after pituitary or other neurosurgery (25), and as part of consultations throughout the hospital. One effect of the COVID-19 pandemic has been the cancellation of most elective pituitary operations, though neurosurgical interventions for subarachnoid haemorrhage or traumatic brain injury continue to induce SIAD. Patients with SIAD will continue to be treated according to well established guidelines (22, 23). As patients who develop SIAD after pituitary surgery characteristically do so after discharge from hospital, it will continue to be important to draw attention to the possible occurrence of hyponatraemia, to both patients and primary care physicians. Hyponatraemia has been recorded to be the commonest cause of hospital readmission following transsphenoidal surgery; as access to routine phlebotomy may be compromised by the needs for social isolation, and the limitations of many health provisions, the emphasis should be on prevention and awareness. **Patients should be advised to limit their fluid intake in the two weeks following surgery, to drink only to thirst and to measure their body weight daily.** We recommend that the primary care physician is contacted if there is weight gain, bloating or unusual headache. Instruction on hyponatraemia-associated symptoms, such as headache, dizziness, nausea or fatigue, is important.
- All other patients with chronic SIAD, who are treated with either fluid restriction, urea or vaptans should be instructed to continue their treatment as usual. We recommend to advise the patients to daily monitor their body weight and to be aware of hyponatraemia symptoms (see above), where access to phlebotomy for electrolytes is restricted.

#### What to look at if patients with hyponatraemia are admitted to the hospital with acute infection with COVID-19

- Hyponatraemia occurs in 30% of pneumonia cases (26), and several studies show that admission hyponatraemia predicts increased mortality and morbidity (27, 28, 29), independent of the underlying disease. Of note, 60% of SARS-COV-1 patients were reported to have mild hyponatraemia (30). Although the prevalence rate of hyponatraemia in COVID-19 is not yet known, currently available data suggest hyponatraemia is uncommon in COVID-19 (31, 32). This may be explained by the high frequency of volume depletion in COVID-19, as a result of the significant increase in insensible fluid losses, and the use of diuretic therapy to treat pulmonary oedema; these factors tend to produce hypernatraemia rather than hyponatraemia.
- As the mortality from hypovolaemic hyponatraemia is higher than that of SIAD (24), the temptation is, that if it does occur in COVID-19 patients, to respond with **i.v. fluid resuscitation.** However, in severely ill COVID-19 patients, clinical experience dictates that **caution should be exercised in the rate of i.v. fluid administration, because of the risk of precipitating pulmonary oedema**. Similar caution is advised in patients with severe hyponatraemia complicated by symptoms of cerebral irritation. Although guidelines recommend bolus hypertonic saline treatment to rapidly elevate plasma sodium concentration (22), and published data suggest that this is effective (33), the sudden volume load might cause pulmonary oedema. In this circumstance, the safer option might be low dose hypertonic saline infusion, with careful control of the volume of fluid administered; the clinical experience in the COVID-19 situation is insufficient to be definitive about this, and each centre should use the method with which they are most familiar.

Table 1 highlights the risks and preventive measures in patients with DI or hyponatraemia in times of COVID-19 and Table 2 shows the management of DI and hyponatraemia in the COVID-19 patient in intensive care.

**Table 1:** Risks and protective measures in DI and hyponatremia in times of COVID-19.

	Risks	Protective measures
Diabetes insipidus	**Dilutational hyponatraemia** as side effect of desmopressin therapy	Delay desmopressin dose once or twice weekly; Advise to regularly control body weight
		Drink to thirst
	**High risk for dysnatraemia if admitted to the hospital** due to missed desmopressin dose, reduced fluid intake, increased insensible losses and the potential need for diuretic therapy	Endocrine consultation for every patient with DI to reduce prescribing errors and to advise with fluid management
		Patient empowerment
		Appropriate stress dosing of corticosteroids in patients with additional ACTH deficiency
Hyponatraemia	**New diagnosis of SIAD** after neurosurgical interventions, brain injury or subarachnoid hemorrhage	Advise to limit fluid intake for two weeks following surgery / injury
		Measure body weight daily and aim to stay at eunatraemic weight
		Drink only to thirst
		Know the early symptoms of hyponatraemia

**Table 2:** Management of DI and hyponatraemia in the COVID-19 patient in intensive care setting.

	Clinical scenario	Action
Diabetes insipidus	Routine care	As per published guidelines Careful attention to desmopressin dose and fluids
	Hypernatraemic dehydration	Hypotonic IV fluids Urine losses + insensible losses = agreed target to reverse hypernatraemia Careful monitoring for ARDS and pulmonary oedema Prophylactic anticoagulation
Hyponatraemia	Routine care	As per published guidelines
	Hypovolaemic hyponatraemia	IV 0.9% sodium chloride solution Rate individualized to patient and agreed with intensivists Careful monitoring for ARDS and pulmonary oedema
	Acute severe hyponatraemia with CNS irritation	Aim to elevate pNa by 8–12 mmol/l/24 h Continuous IV infusion of 3% saline recommended to control fluid load or careful IV bolus 3% saline Monitor pNa every 2–4 h

AKI, acute kidney injury; pNa, plasma sodium concentration.

In summary, patients with central DI or hyponatraemia should be managed according to existing guidelines as ambulatory patients, with careful explanation to DI patients of how to avoid dilutional hyponatraemia in circumstances where plasma sodium measurements are difficult to access. Patients with DI are vulnerable to adverse effects and potentially poor outcomes if hospitalized with COVID-19. Hypernatraemia seems to be a common problem of COVID-19 in intensive care units, most probably due to insensible water losses from the constant pyrexia, and due to increased respiration rate. This tendency may be enhanced in patients with DI, who are at risk of hypernatraemic dehydration. Treatment of dehydration must be tempered with the potential to precipitate pulmonary oedema with i.v. fluid; treatment plans should be carefully agreed between endocrinologists and intensivists. Hyponatraemia in severely COVID-19 affected patients should be treated by guideline principles, but with similar caution with respect to the volume of i.v. fluids in hypovolaemic hyponatraemia, or in acute hyponatraemia with cerebral irritation.

## Disclaimer

Due to the emerging nature of the COVID-19 crisis this document is not based on extensive systematic review or meta-analysis, but on rapid expert consensus. The document should be considered as guidance only; it is not intended to determine an absolute standard of medical care. Healthcare staff need to consider individual circumstances when devising the management plan for a specific patient.

## Declaration of interest

The authors declare that there is no conflict of interest that could be perceived as prejudicing the impartiality of this guidance.

## Funding

This guidance did not receive any specific grant from any funding agency in the public, commercial or not-for-profit sector.
